# The positive impact of a facilitated peer mentoring program on academic skills of women faculty

**DOI:** 10.1186/1472-6920-12-14

**Published:** 2012-03-23

**Authors:** Prathibha Varkey, Aminah Jatoi, Amy Williams, Anita Mayer, Marcia Ko, Julia Files, Janis Blair, Sharonne Hayes

**Affiliations:** 1 Division of Preventive and Occupational Medicine and Department of Medicine, Mayo Clinic, Rochester, MN, USA; 2 Department of Oncology at Mayo Clinic, Rochester, MN, USA; 3 Division of Nephrology and Department of Medicine, Mayo Clinic, Rochester, Rochester, MN, USA; 4 Division of Primary Care Internal Medicine at Mayo Clinic, Scottsdale, AZ, USA; 5 Department of Medicine, Women's Health Center at Mayo Clinic, Scottsdale, AZ, USA; 6 Division of Infectious Diseases at Mayo Clinic, Scottsdale, AZ, USA; 7 Division of Cardiovascular Diseases at Mayo Clinic, Rochester, MN, USA

## Abstract

**Background:**

In academic medicine, women physicians lag behind their male counterparts in advancement and promotion to leadership positions. Lack of mentoring, among other factors, has been reported to contribute to this disparity. Peer mentoring has been reported as a successful alternative to the dyadic mentoring model for women interested in improving their academic productivity. We describe a facilitated peer mentoring program in our institution's department of medicine.

**Methods:**

Nineteen women enrolled in the program were divided into 5 groups. Each group had an assigned facilitator. Members of the respective groups met together with their facilitators at regular intervals during the 12 months of the project. A pre- and post-program evaluation consisting of a 25-item self-assessment of academic skills, self-efficacy, and academic career satisfaction was administered to each participant.

**Results:**

At the end of 12 months, a total of 9 manuscripts were submitted to peer-reviewed journals, 6 of which are in press or have been published, and another 2 of which have been invited to be revised and resubmitted. At the end of the program, participants reported an increase in their satisfaction with academic achievement (mean score increase, 2.32 to 3.63; *P *= 0.0001), improvement in skills necessary to effectively search the medical literature (mean score increase, 3.32 to 4.05; *P *= 0.0009), an improvement in their ability to write a comprehensive review article (mean score increase, 2.89 to 3.63; *P *= 0.0017), and an improvement in their ability to critically evaluate the medical literature (mean score increased from 3.11 to 3.89; *P *= 0.0008).

**Conclusions:**

This facilitated peer mentoring program demonstrated a positive impact on the academic skills and manuscript writing for junior women faculty. This 1-year program required minimal institutional resources, and suggests a need for further study of this and other mentoring programs for women faculty.

## Background

Women physicians continue to lag behind their male counterparts in academic advancement and in promotion to leadership positions [1]. Women have constituted almost 30% of students admitted to medical school as far back as 1980, and since 2003 men and women have been admitted to medical school in virtually equal numbers [2,3]. In 1980, 9% of female faculty achieved the rank of full professor during their academic career. The Association of American Medical Colleges 2010 benchmarking report on women in leadership revealed this percentage had improved only modestly to12.5%. This is compared to 30% of all male faculty achieving full professor status in 2010, a stable proportion over the same 30 years [1].

Statistics regarding women faculty in leadership positions are equally disconcerting. In 2010, only 13% of department chairs were female, with numerous medical schools reporting never having had a woman in a department chair or dean position [2,3]. Women faculty's failure to make substantial gains in academic rank and leadership is even more alarming given the attention and efforts to correct this imbalance for over 20 years [4-19]. Lack of mentoring, demands of clinical practice, and family obligations, as well as poor or absent succession planning, have been identified as possible contributors to these disparities [5,6,20-28].

Mentoring has long been an essential component of career advancement within academic medicine and other disciplines [5-10]. Understanding the important role of mentoring has led many academic medical centers to develop, support, and promote mentoring programs. The mentors, mentees, and the programs themselves are challenged by increasing clinical, research, and administrative demands [27,29,30]. Beyond the traditional dyadic mentoring model, a number of mentoring models, including multiple- and peer-mentoring models, have been described and studied [31-36]. Peer mentoring, in a variety of permutations, has been described as an alternative to the traditional dyadic mentoring model [33-35,37-39]. In facilitated peer mentoring programs, faculty typically work collaboratively in groups of 3 to 5 with other faculty who are of similar rank and who have similar academic interests. A facilitator (a faculty member of a higher academic rank) works with the group in meeting their scholarly goals. Very little has been written on the actual outcomes of such nontraditional mentoring models on academic skills and scholarly output of mentees.

Reports of women faculty's greater appreciation of the process of collaboration [40], and differences in preferred work style [41], may lead to a broader applicability of peer mentoring models with women than their male colleagues. Women have reported more difficulty in identifying mentors and developing successful mentoring relationships. Whether gender concordant mentors are of benefit has been a point of dispute [29].

The Department of Medicine at Mayo Clinic, Rochester, Minnesota, has 153 women (24% of total) faculty. Gender disparities in academic rank and leadership opportunities led the department's leadership team to evaluate strategies to close the gap. Focus group discussions and surveys suggested that a lack of mentoring and social isolation were common themes reported by women faculty. Peer mentoring was considered to be a critical ingredient to a multipronged approach to address these issues. By means of this paper, we aim to discuss the process and outcomes of an expanded 12-month facilitated peer mentoring project. Our primary objective was to study the impact of facilitated peer mentoring on scholarly output, specifically manuscripts submitted for publication. Secondary objectives were to study the impact of peer mentoring on self-efficacy of writing skills and networking.

## Methods

This project was declared exempt by the Mayo Clinic Institutional Review Board.

### Formation of peer mentoring groups

All women faculty in the Department of Medicine at Mayo Clinic, Rochester, Minnesota, who hold the rank of instructor or assistant professor (*n *= 106) were invited to participate in the project and asked to submit names of peers of similar academic rank with whom they wished to potentially work in a group setting.

Twenty-five of these instructors or assistant professors expressed preliminary interest in this project. Eight registered but subsequently deferred participation shortly after the teams were formed; hence, a total of 19 mentees participated in this program and formed the study sample for this project. A preventive medicine fellow and a nurse practitioner who had heard of the program and had been invited by enrolled faculty were included in the group of 19 mentees.

These mentees were divided into 5 peer groups. Mentees who had signed up for the project were grouped by the organizers of the program to optimally match research and clinical interests based on their curriculum vitae and scholarly interests. Although we did not formally collect information on whether they had academic mentors prior to the start of the program, based on informal discussions, most, if not all, did not have mentors.

Four women faculty with extensive experience in faculty development and mentoring who had achieved the rank of associate professor or professor served as facilitators for the groups; one served as the facilitator for 2 groups. These 4 women chose to serve voluntarily and were chosen based on recommendations of leaders in the department of medicine. At enrollment, facilitators and participants signed a good-faith agreement to remain active and engaged in their group.

#### Orientation

The kickoff for this program was a 1-day workshop. The first half of the orientation was designed for the facilitators, during which overall strategies and program goals, group process, and problem solving were discussed. Topics included not only traditional "mentor" expectations such as support roles and career advising, but also discussions on how to deal with specific issues and problems previously encountered by facilitators, such as conflict among group members, work-life balance, and illness and maternity issues. The mentees joined the group for the second half of the orientation workshop, and the program purpose, goals, processes, and expectations were outlined. In addition, a baseline 25-item self-assessment was administered by means of which academic skills, self-efficacy, and academic career satisfaction were measured using a Likert scale, ranking from 1 for "strongly disagree" to 5 for "strongly agree."

#### Small group work

During the orientation session, each group discussed and came to agreement on processes and logistics. They also developed the project charter, optimal meeting times, and frequency, and identified educational skills and informational needs required to accomplish goals. To allow flexibility with scheduling and venue (e.g.face to face, at work or off campus, virtual), the groups were not required to meet on a specific day. A tentative 12-month timeline of projects, primary authors for the manuscripts, and target journals for manuscript submission was also developed.

#### Program execution

During the year-long program, facilitators were encouraged to meet regularly with their groups at intervals of every 2 to 4 weeks, participate in brainstorming sessions, review the suitability of topics for development and manuscript submission, edit drafts, and provide additional individual and project mentoring on an ad hoc basis. Additionally, phone conferences were scheduled for each month among the 4 facilitators.

#### Program assessment

At the end of the program, the initial self-assessment survey was repeated, with the addition of several questions allowing free-text response options specifically aimed to assess the value and effectiveness of the program to participants. A paired *t*-test was used to compare serial survey results, and a *P *value of < 0.05 was considered statistically significant. The qualitative comments were reviewed and categorized into either *positive/value added *or *constructive/suggested improvement *comments.

## Results

Five peer mentoring groups, comprising a total of 19 as mentees and 4 as facilitators, formed the project group. Of the 19 mentees, 17 were on faculty in the Department of Medicine of Mayo Clinic, Rochester, Minnesota. The mean number of years on faculty was 6.20 (range, 1.5-22 years). Six of the mentees held an academic rank of assistant professor, 11 had achieved the academic rank of instructor, and the clinical fellow and nurse practitioner had no academic rank. The participants were divided into 5 groups each with 3 to 5 mentees and 1 facilitator.

Groups were encouraged to meet once weekly, and to meet at least monthly with their facilitator. The actual meeting frequency varied widely from every week to once every 4 to 6 weeks. In one group, the facilitator joined the mentees for most meetings. Others met with their mentees at least once a month. Regardless of meeting frequency, mentees had regular e-mail and phone connectivity with other mentees and facilitators between meeting times. The facilitators had a monthly in-person or phone meeting during which they updated the others, discussed successes, problems and roadblocks, and shared best practices.

At the end of the 12-month project, 9 manuscripts were submitted (mean/group 1.25, SD 0.50, range 1-3) by the 5 groups. Of the 9 submitted, 6 papers are in press or have been published and another 2 have been invited to be revised and resubmitted. In addition, 1 peer mentoring subspecialty group designed and completed a clinical research project, and at the time of manuscript writing had presented their findings at a national meeting. They are working on completing the manuscript related to this project. The distribution of papers and academic work completed was not equal between the groups: 1 group completed 3 manuscripts, 3 groups completed2 manuscripts, and 1 group completed1 manuscript. Another group did not focus on manuscript writing, but on clinical protocol design and execution. In addition, 1 of the groups who completed 2 manuscripts also completed and submitted a grant.

Although each mentor and participant signed an agreement to consistently participate in the group mentoring sessions, the individual groups were not asked to commit to a specific frequency of meetings and were not required to document number and length of interactions. Thus, data on the distribution of frequency or total number of hours the groups met are not available.

Participation in the peer mentoring program was associated with the achievement of several additional academic and career goals. Among these participants were 2 individuals who pursued advanced degrees: a masters of public health and a masters in academic medicine, and the appointment of 1 individual as a divisional practice chair. Four individuals received 5 grants (4 institutional grants and 1 position on a KL2 grant). These achievements were distributed throughout the groups without concentration in the groups with the higher manuscript completion.

Self-efficacy and career goal data were obtained from the study participants at baseline and after the study period through the self-assessment survey and is represented in Table 1. All 19 participants completed the baseline survey, while 17 completed the post-study survey. The 2 participants who did not complete the post-study survey were absent at the time the survey was conducted. At the start of the project, most study participants were not satisfied with their academic rank (mean, 2.00, range, 1-5), and were interested in participating in a collaborative research project (mean, 4.16) to improve their academic rank and skills. Another identified need of the study participants included a strong interest in developing skills to become an effective mentor (mean score 4.67, range 3-5).

**Table 1:** 25-Item self-assessment survey: characteristics of participants at baseline and at completion of the peer mentoring program

	Questions	Pre-Event**		Post-Event**		Paired *T *test P-value
			
		Mean	± SD*	Mean	± SD	
1	I am satisfied with my current academic rank.	2	1.29	2.32	1.16	0.3163

2	I am satisfied with my academic accomplishments.	2.32	1.11	3.63	0.68	0.0001

3	I wish to be involved in academic projects but lack the skills to be successful.	3.37	1.01	2.42	1.07	0.0083

4	I have a career goal.	4.05	0.71	4.26	0.65	0.1628

5	I have identified specific plans to achieve my career goals.	3.37	0.83	3.78	0.94	0.007

6	I have the time to attend after-hours seminars to enhance my academic skills.	2.74	1.28	2.63	1.54	0.7162

7	I have the skills necessary to effectively search the medical literature.	3.32	1.06	4.05	0.85	0.0009*

8	I am familiar with the Office of Scientific Publications.	2.79	1.18	3.68	0.75	0.0057*

9	I understand the services offered by the Protocol Development Office.	1.74	0.93	2.37	0.96	0.0239*

10	I am satisfied with my ability to use EndNote as a toll for managing references.	2.26	1.28	3.47	1.22	0.0017*

11	I have the skills necessary to take a clinical question and develop a clinical research project.	3.58	1.07	3.84	1.01	0.3496

12	I understand the process for submitting a CR20.	2.63	1.3	3	1.2	0.0491*

13	I am satisfied with my ability to effectively use Power Point.	3.63	0.68	4.37	0.76	0.0001*

14	I have identified an effective academic mentor.	2.78	1.35	3.68	1.49	0.0737

15	I would prefer to have a same-gender mentor.	3.11	1.1	2.95	1.13	0.5778

16	I know how to apply for academic rank.	3.37	1.3	3.94	0.94	0.0229*

17	I feel confident in my ability to assist residents in designing, completing, and publishing academic projects.	2.56	1.1	3.21	1.03	0.0137*

18	I would like to become an effective mentor.	4.67	0.59	4.58	0.77	0.1631

19	I am an effective public speaker.	3.42	1.02	3.53	0.96	0.5778

20	I would benefit from training in public speaking.	3.37	0.9	3.79	0.63	0.1341

21	I would be interested in participating in a collaborative research project.	4.16	0.69	4.5	0.62	0.0827

22	I have the skills necessary to write a comprehensive review paper.	2.89	1.15	3.63	0.96	0.0017*

23	I can critically evaluate the medical literature.	3.11	1.15	3.89	0.88	0.0008*

24	I am satisfied with my ability to effectively network with other physicians in this institution to find opportunities for collaboration.	2.78	0.94	3.58	1.02	0.0027*

25	I know how to find a good mentor.	2.79	1.03	3.42	1.17	0.0419*

	**Post-Event Survey Only Questions**					
	
	Participation in the peer mentoring program has allowed me to come nearer to accomplishing my academic career goal(s).			3.78	1.03	N/A
	
	Would you recommend the peer mentoring program to a colleague? Yes or No				16-Yes 89% 1 did not answer	

*P values that are statistically significant.

** Scale: 1, Strongly Disagree; 2, Somewhat Disagree; 3, Neutral; 4, Somewhat Agree; 5, Strongly Agree.

At the conclusion of the mentoring program, participants reported an increase in their satisfaction with academic achievement (mean score increase, 2.32 to 3.63; *P *= 0.0001) and improved skills necessary for academic success (*P *= 0.0083). As shown in Table 1, participants also reported an improvement in skills necessary to effectively search the medical literature (mean score increase, 3.32 to 4.05; *P *= 0.0009); use EndNote to manage references (mean score increase, 2.26 to 3.47; *P *= 0.0017); write a comprehensive review article (mean score increase, 2.89 to 3.63; *P *= 0.0017); and critically evaluate the medical literature (mean score increase, 3.11 to 3.89; *P *= 0.0008). Skills related to developing clinical research projects from original clinical questions were not enhanced following participation in the program (mean score increase, 3.58 to 3.84; *P *= 0.3496), whereas understanding the process for submitting research protocols for institutional funding was gained (mean score increase, 2.63 to 3; *P *= 0.049). Study participants also stated that the confidence in their ability to assist residents in designing, completing, and publishing academic projects improved over the course of the program (mean score increase, 2.56 to 3.21, *P *= 0.014). At the conclusion of the 12-month study period, there was also a significant improvement in perceived ability to effectively network with other physicians in the institution to find opportunities for collaboration (2.78 vs. 3.58, *P *= 0.0027) and an improved understanding of how to identify an effective mentor (2.79 vs. 3.42, *P *= 0.0419). Qualitative feedback from participants is noted in Table 2.

**Table 2:** Post-program comments from mentored participants

Favorable	Constructive
"Working with colleagues toward goals seems very intellectually and professionally satisfying."	"...I think the time frame was too short given data collection obstacles."

"2 more publications"	"Difficult to find time to meet which makes sharing the load challenging."

"Having a caring, motivating and brilliant mentor has been the best part of this program for me."	"Personally, I think that it is better to try to have true shared interests and directions with peer mentors."

"Helped develop skills in research leading to publication; allowed networking within department."	".... hard to get everyone together, hard to find topics"

"I have more confidence about writing a paper and using available resources to do so."	"Our group had too many diverse interests which made it difficult to gain momentum on any particular writing topic..."

"Promotes collegiality, promotes completion of projects by ensuring accountability/providing motivation...."	"...I would try to encourage some type of protected time (if available)."

"Motivates you to accomplish other goals... projects, academic appt. etc."	
	
"I'd recommend it. It helps foster the team-spirit and a long-term relationship if the mentor and mentee find it a good fit."	
	
"Potential for success! Relationship building..."	
	
"Especially for those new to the institution... allows introduction to broad scope of resources available here and networking"	
	
"Excellent mentoring accountability to peer & mentors. I liked the women-only focus."	

The facilitators were highly satisfied with the peer mentoring program (mean satisfaction score 4.4, SD 0.55; scale range 1-5) and with the overall interaction of their individual peer mentoring groups (mean 4.8, SD 0.45; scale range 1-5). The facilitators' satisfaction with the quality of the papers was also high, with a mean satisfaction score of 4.25 (SD 0.50; scale range 1-5). All facilitators and 89% of the peer participants reported that they were willing to both facilitate future peer mentoring groups and recommend the program to other faculty members.

## Discussion

A recent call for cultural change acknowledged the disappointing progress in the advancement of women in academic medicine [3]. This project was intended to modify reported lack of effective mentoring for women faculty and therefore maximize academic opportunities for women in the lower academic ranks. The results of this 1-year effort led to 9 manuscripts (6 of which at the time of this report are either in press or published) and 5 institutional grants. Moreover, when surveyed, participants reported improvements in their career satisfaction, a higher level of engagement in academic pursuits, and progress in formulating specific plans relevant to achieving career goals. Even with respect to acquiring specific skill sets, such as skills with PowerPoint software and other tools, comprehensively reviewing the medical literature, identifying funding opportunities, and learning how to apply for academic promotion, the peer participants reported gains based on pre- and post-survey results. Based on tangible evidence of accomplishment, such as publications and grant funding, as well as participant surveys, this project was deemed a success and has now been expanded to all male and female junior faculty in the department of medicine.

Peer mentoring, as used in this project, has been described as an effective mentoring method for both male and female faculty [15,16,19-21]. At the present time, little published data exist regarding the scholarly output of mentees enrolled in such models to determine whether gender differences will be seen in academic productivity and advancement. One clue in predicting success may be the collaborative underpinnings of most peer mentoring approaches. A recent qualitative study of faculty views on collaboration revealed that women appreciate the process of collaboration in and of itself, independent of the collaborative product. Male faculty reported an interest in collaboration only as a means to the end result, or academic product [40].

In our facilitated peer mentoring program, participants were, by design, required to work in a collaborative manner within their own peer group. Also, when queried, the mentees reported an improvement in their ability to effectively network with other physicians to find opportunities for collaboration. Our questions, as posed, did not delineate participants reporting an improvement in networking skills in isolation; rather, it was asked in the context of networking as a way to find opportunities for collaboration.

Our program also explored the efficacy of a peer-to-peer mentoring group guided by a more senior faculty member who was gender-matched and acted as a facilitator. The peer mentoring groups in this study were loosely constructed around 3 models of collaboration described by Schneider [22,29]. Our intent had been to require the peers to self-identify potential peers and, hence, groups by chosen interest. Groups were constructed to reflect shared values, interests, and skill sets to as great an extent as possible. This highly structured approach, which allowed peers to choose their peer mentoring groups but did not require them to find their own mentor, may have accounted for the successful outcomes of this project. Moreover, the fact that the facilitators served on a voluntary basis and were specifically chosen for this purpose may have led to a highly conducive environment that promoted academic accomplishment among women faculty.

There are a number of possible explanations for the variable outcomes among the mentoring groups. Facilitator and participant engagement, discrepancies in baseline academic skills, and frequency of group interactions all could have played a role in the degree of each group's success. As above, the facilitators and participants were not required to log meeting times or report on the degree of individual engagement, but all participants did sign a good-faith agreement to remain active participants. Of note, the group that completed only 1 manuscript was comprised of individuals from different specialty divisions, whereas the other groups included individuals from the same specialty or division. This diversity in background may have led to a slower ramp-up period for this group.

In response to the survey item, "I have the skills necessary to take a clinical question and develop a clinical research project," peer participants' responses did not show a statistically significant improvement over time. This result was not unexpected, perhaps because the project was designed to enhance the writing skills of the peer groups by producing clinical review articles or resurrecting and publishing results from a previously acquired data set. The relatively limited 12-month time frame prompted a stronger emphasis on publishing, which constitutes a solid criterion for academic promotion at most academic medical centers, and less emphasis on idea generation and research project initiation.

As in traditional mentoring relationships, although the gist of instruction regarding manuscript writing was likely similar across all groups, the timing and method of instruction would have varied based on immediacy of issues being discussed in the group, the content of the manuscript, the baseline knowledge of the group members, and the interaction between the facilitators and mentees. Item 14 on the assessment survey, "I have identified an effective mentor," showed a nonsignificant improvement at the end of the program; we are unsure if this means that the various groups were dissatisfied with the mentor assigned to them or if this reflects the participant's response to finding an effective traditional one-on-one mentor.

This program was not designed to serve the facilitators in any way; there were, however, unexpected benefits from their perspective. These included the opportunity to interact with motivated early-career faculty, to participate in a novel and exciting department-wide program, and to serve as coauthor on papers with a variety of fresh topics. Thus, this new program itself was truly bidirectional in its benefits, providing positive effects among both participants and facilitators.

This study is not without limitations. First, although this study spanned a full year, we do not have data on long-term outcomes beyond the project year. It remains to be seen whether the favorable effects observed here are in fact sustained and whether they ultimately lead to academic promotion and increased leadership roles among these women faculty. Secondly, in an effort to be evenhanded in allocating opportunities, this facilitated peer mentoring intervention was provided to all interested junior women faculty within the department of medicine. Traditional one-on-one mentoring continues to be the mainstay for mentoring models in the department, and this project was introduced to engage faculty without such mentors, which likely accounted for the lower number of women who signed up for the program. We did not compare the academic achievements of the faculty who did not sign up for the program with those who did, as this was beyond the scope of the study. Third, in the absence of a comparative arm that might have included either another intervention or no intervention, it is impossible to assess accurately the true positive impact of this facilitated peer mentoring approach. It remains possible that the program self-selected for highly motivated individuals who might have been productive independent of their participation in the program. We point out that this third criticism could be raised regardless of details on the participants or specifics related to the program, and that it remains a criticism of any mentoring program that does not include a control group. Nevertheless, we also point out that this program's heavy emphasis on introducing the mentees to the practical aspects of acquiring clinical research skills, writing papers, and getting these papers published likely had a direct effect on the favorable survey and productivity outcomes described here.

Future efforts may include extending the duration of the intervention, assess long-term outcomes, and seek comparative data that might result from studying other approaches. Although the peer participants in our women-only program did not strongly advocate same-gender mentorship, future studies might also focus on whether different mentoring approaches might resonate better with some groups versus others based on gender distribution.

## Conclusion

Mentors are a valuable resource within academic medical centers, and the spirit of altruism that drives such mentoring also drives the academic accomplishment of faculty at most academic medical centers. The current project extended such mentoring opportunities to junior women faculty, demonstrated preliminary positive outcomes in terms of academic accomplishment, and suggests a need for further study of this and other mentoring programs for women faculty.

## Competing interests

The authors declare that they have no competing interests.

## Authors' contributions

All authors read and approved the final manuscript; all authors contributed to study design, results analysis and write up of the manuscript.

## Pre-publication history

The pre-publication history for this paper can be accessed here:

http://www.biomedcentral.com/1472-6920/12/14/prepub
